# Experiences From the First Year of Using Garadacimab to Treat Hereditary Angioedema and Acquired Angioedema

**DOI:** 10.1002/clt2.70205

**Published:** 2026-10-11

**Authors:** Markus Magerl, Kathrin Schön, Janine Ruhe, Felix Aulenbacher, Leonie Shirin Herzog, Thomas Buttgereit

**Affiliations:** ^1^ Angioedema Center of Reference and Excellence (ACARE), Institute of Allergology Charité – University Medical Center Berlin, Corporate Member of Freie Universität Berlin and Humboldt‐Universität zu Berlin Berlin Germany; ^2^ Fraunhofer Institute for Translational Medicine and Pharmacology ITMP Immunology and Allergology Berlin Germany

**Keywords:** C1 inhibitor deficiency, disease control, garadacimab, HAE, long‐term prophylaxis, normalization of life, quality of life

## Abstract

**Background:**

Garadacimab, a first‐in‐class, recombinant monoclonal antibody targeting activated FXII, is available in Germany for long‐term prophylaxis (LTP) of hereditary angioedema (HAE) since March 2025. We aimed to examine garadacimab real‐life treatment outcomes at our Berlin Angioedema Center of Reference and Excellence (ACARE), the reasons for switching therapies, and approaches to tailoring treatment individually.

**Methods:**

In total, 29 patients (27 patients with HAE and 2 patients with angioedema due to acquired C1 inhibitor deficiency) were treated in regular care with garadacimab; 3 patients discontinued garadacimab due to lack of efficacy after loading dose or second dose and were excluded from analysis due to insufficient follow up. Ultimately, 26 patients were monitored for 26 weeks (median) using the angioedema control test (AECT) and the angioedema quality of life questionnaire (AE‐QoL). In seven patients, garadacimab from the open‐label‐VANGUARD study was continued in regular care. Patients who had previously participated in the study—provided they were symptom‐free—were offered the option, upon transitioning to routine care, to extend the interval between injections by alternating between three and four days. Patients who were switched to garadacimab in routine care were offered the same option if they were completely symptom‐free 2 months after starting the therapy.

**Results:**

Overall, 16 patients switched from previous LTP to garadacimab mostly at the end of their individual treatment interval and due to the prospect of higher efficacy and convenience. The injection interval could be extended in most patients (median, 42 days, range 31–56). The mean AECT and AE‐QoL improved clinically meaningfully, regardless of previous treatment (9.2–14.4 and 35.5–14.9 points, respectively), however, improvement in patients switched from previous LTP was less pronounced when compared to patients that switched from on‐demand treatment (11.2–14.5 vs. 6.7–14.2 points and 29.4–14.3 vs. 43.1–15.7 points, respectively). No side effects were reported.

**Conclusions:**

Garadacimab showed high efficacy and safety and the gradual extension of injection intervals presented underline its potential for longer dosing intervals that may further reduce treatment burden.

**Abbreviations**
AAE: Acquired angioedema
ACARE: Angioedema Centers of Reference and Excellence
AE: angioedema
AE‐QoL: Angioedema Quality of Life Questionnaire
AECT: Angioedema Control Test
BK: Bradykinin
C1INH: C1‐inhibitor
CARE: Chronic Angioedema Registry
CSU: Chronic spontaneous urticaria
FXII: Factor XII
HAE: Hereditary angioedema
HAE‐nC1INH: Hereditary Angioedema with normal C1 Inhibitor
LTP: Long term prophylaxis
MCID: Minimal clinically important difference
MYOF: Myoferlin
ODT: On‐demand treatment
QoL: Quality of Life
UNK: Unknown mechanism/mutation

## 1 Introduction

Hereditary angioedema (HAE) is a rare autosomal‐dominant inherited disease characterized by recurrent, disfiguring and painful swelling attacks in several parts of the body, including life‐threatening laryngeal attacks [1]. In most cases the disease is caused by mutations in the SERPING1 gene that lead to deficiency of C1 Inhibitor (C1INH) resulting in uncontrolled release of bradykinin (BK) with increased vascular permeability and subsequent plasma extravasation [2, 3]. In more rare cases C1INH deficiency is acquired, that is angioedema due to acquired C1INH deficiency (AAE‐C1INH), which often is associated with lymphoproliferative disease or autoimmune disease [4].

In recent years more rare subtypes of HAE, that is HAE with normal C1 Inhibitor (HAE‐nC1INH), have been discovered. There are some genetic variants described in HAE‐nC1INH that affect genes involved in the Kallikrein‐Kinin‐System causing bradykinin‐mediated angioedema (AE‐BK), for example factor XII (HAE‐FXII), while other variants in genes are considered to cause vascular endothelial dysfunction, for example mutations in myoferlin (HAE‐MYOF) [5]. In very rare cases the cause remains unknown (HAE‐UNK) [6].

The occurrence of HAE attacks usually is unpredictable, but also certain trigger factors such as mechanical trauma, surgery, exercise, emotional stress, puberty or certain medication are described. Moreover, some patients experience prodromal signs and symptoms preceding attacks [7].

Patients with uncontrolled HAE often experience a significant reduction in their disease‐related quality of life (QoL) [8]. The treatment goals formulated in the current guidelines for the management of HAE are to achieve complete disease control and to normalize patients' lives [9]. At present, this goal can mainly be achieved through long‐term prophylaxis (LTP), that is, the continuous use of medication to prevent attacks [1]. Moreover, there are also options for on‐demand therapy (ODT), which include recombinant or plasma‐derived (pd) C1INH replacement, the bradykinin B2 receptor antagonist icatibant, and more recently, the oral plasma kallikrein inhibitor sebetralstat [1, 10].

Various treatment options for modern LTP have been available for several years or were recently approved for HAE, among them plasma‐derived C1INH replacement (intravenously or subcutaneously every 3–4 days), the monoclonal antibody against plasma kallikrein lanadelumab (subcutaneously every 2–4 weeks), the small molecule inhibiting plasma kallikrein berotralstat (once daily 1 tablet) and the antisense oligonucleotide donidalorsen (subcutaneously every month or every 2 months) [11, 12, 13].

In March 2025, garadacimab—a first‐in‐class recombinant monoclonal antibody that targets activated FXII (FXIIa) and thereby inhibits the activation of the contact system and the subsequent formation of bradykinin—became available in Germany. Garadacimab is approved for LTP of HAE attacks in patients aged 12 and older. In the VANGUARD Phase‐3 study, garadacimab 200 mg dosed once a month showed high efficacy over placebo and was well‐tolerated [14]. As of now, its effectiveness and safety remain unknown in larger cohorts in real‐life including patients with AAE‐C1INH and HAE‐nC1INH. Moreover, it remains unclear why patients being on existing treatment switch, how a switch is managed, and if garadacimab injection intervals can be individually adapted when patients remain attack free as previously shown with other LTP [15, 16].

Here, we report our first‐year real life experience with the efficacy and tolerability of garadacimab in patients with HAE and AAE‐C1INH at the Angioedema Center of Reference and Excellence (ACARE), Charité Berlin [17].

## 2 Methods

### 2.1 Patient Population

At the time of the data cut (March 19, 2026), a total of 29 patients (17 female, median age 41 years, range 17–84) have received treatment with garadacimab in routine care. Twenty‐four patients were diagnosed with HAE‐C1INH (median age 41 years, range 17–84 years, standard deviation [SD] 17.8), two had AAE‐C1INH (64 and 79 years), one patient each had HAE‐FXII (female, age 57 years), HAE‐MYOF (female, age 68 years) and HAE‐UNK (male, age 62 years). The mean period from onset of symptoms to diagnosis of HAE and AAE‐C1INH in the cohort was 11.3 ± 14 years and 3 ± 4.2 years, respectively. In 93% of HAE patients a positive family history was known.

Prior to switching to garadacimab, 16 patients (55%) had received other LTP for a median treatment period of 38 months (range 1–125 months): 10 patients with HAE‐C1INH had used lanadelumab (median treatment period 50 months, range 17–125 months) and two had used pdC1INH (median treatment period 42 months, range 36–48 months). Two patients with AAE‐C1INH had used lanadelumab (60 months) and pdC1INH (3 months), respectively. Furthermore, one patient with HAE‐MYOF switched from tranexamic acid (one month of treatment), and one patient with HAE‐C1INH switched from berotralstat (14 months of treatment).

Six patients had exclusively used ODT (with icatibant) before the switch and seven patients were direct roll‐overs (RO) from the open‐label extension (OLE) VANGUARD Study (VANGUARD OLE‐RO). (Figure 1).

**FIGURE 1:**
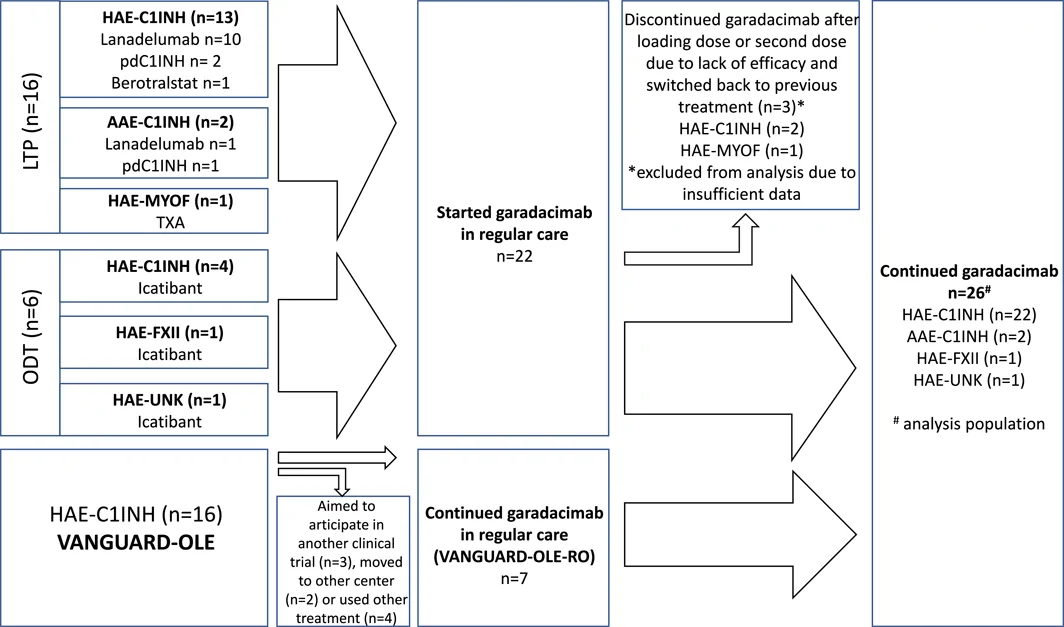
Study flow of patients treated with garadacimab in regular care in the angioedema center of reference of excellence (ACARE) in Berlin until data cut 19 March 2026. HAE‐C1INH: Hereditary angioedema due to C1 inhibitor deficiency; HAE‐FXII: HAE due to mutation in factor XII; HAE‐MYOF: HAE due to myoferlin mutation; HAE‐UNK: HAE due to unknown mechanism; LTP: long‐term prophylaxis; *n*: number; ODT: on‐demand treatment; VANGUARD‐OLE‐RO: Patients from the open label extension (OLE) VANGUARD study that rolled over (RO) to regular care.

Among patients for whom data were available, the median frequency of attacks prior to switching to garadacimab was 2 (range 0–15, *n* = 27) attacks per month, and the median Angioedema Control Test (AECT) score was 10 (range 1–16, *n* = 23) points.

LTP with garadacimab (or its continuation from VANGUARD OLE‐RO) was initiated in all patients between March 04, 2025 and December 20, 2025. The analysis population for garadacimab outcomes included a total of 26 patients; 3 patients were excluded from this analysis due early discontinuation due to lack of efficacy after loading dose or second dose (see also chapter results). All effectiveness and safety analysis were performed collectively in order, first, to obtain a comprehensive picture and, second, due to the low number of cases for patients with AAE‐C1INH and HAE‐nC1INH. The medical history of the patients and safety data of garadacimab was obtained by the Chronic Angioedema Registry (CARE) and complemented by patient records. CARE was approved by Ethics Committee of Charité—Universitätsmedizin Berlin in August 2023 (EA2/145/23).

### 2.2 Treatment and Procedure for Extension of Injection Intervals

In patients treated in regular care, the decision to initiate or to switch to LTP with garadacimab was made on individual basis by shared decision making [18]. No predefined clinical or reimbursement criteria were applied, however, patient reported outcomes for disease activity, individual disease control (AECT) and/or disease‐specific quality of life (AE‐QoL) as well as treatment burden of prior treatment were considered for the switch. The overarching goal of the treatment switch was to achieve complete disease control and to normalize patients' life including reducing the burden of therapy [19]. The initiation of garadacimab took place after providing patients with information about the potential risks and expected benefits in the context of regular care during a consultation. Patients who decided to start or continue therapy with garadacimab were offered the option of individually optimizing (i.e., extending) their treatment interval. The advantages and disadvantages of this approach were discussed with each patient; in particular, the likely occurrence of HAE/AAE symptoms during the extension phase was addressed. Patients who rejected this approach or had concerns received their treatment in accordance with the prescribing information at the approved and recommended dosage (200 mg once a month). For patients already receiving LTP, treatment with garadacimab intended to be initiated on the day the next dose of their current LTP was due. In garadacimab naïve patients, we started with the recommended loading dose of 2 × 200 mg garadacimab, the next injection (200 mg) was administered by the patients themselves 31 days (= 1 month) later (Figure 2). If tolerability and efficiency of garadacimab treatment was well and no signs and symptoms of HAE (prodromal signs and/or attacks) were reported by the patient, we suggested extending the dosing interval stepwise by alternating increases of 3 and 4 days, that is after day 35, 38, 42 etc. This strategy was based on a pre‐defined protocol derived from pharmacokinetic data [20] and our own experience with the use of lanadelumab [15, 16]. The alternation extension by 3 and 4 days had practical considerations: Every second increase in the dosing interval occurs on a weekly basis, resulting in fixed days of the week. A follow‐up visit was scheduled after 3 months shortly before the fourth injection. Until this visit, patients were closely monitored and the continuation of the prolongation of the injection interval (day 45, 48 and 52) was recommended, when they continued being completely symptom free. In the event of HAE signs/symptoms, patients treated themselves with ODT, the individual injection interval prolongation was stopped and the injection intervals were shortened to the penultimate interval that had provided freedom from signs/symptoms. In VANGUARD OLE‐RO patients, the indication for LTP was assumed to persist, and continuation was suggested by the treating physician. The VANGUARD OLE‐RO patients completed the trial and have been symptom‐free for months or years. Prior to the first injection after the end of the trial, these patients were asked to extend the injection interval according to the following protocol (Figure 2): We prolonged the monthly (= 31 days) dosing interval of garadacimab 200 mg injections stepwise by alternating increases of 3 and 4 days, that is after day 35, 38, 42 etc. A follow‐up visit was scheduled after 3 months shortly before the fourth dosing‐interval step. Until this visit, patients were closely monitored and the continuation of the prolongation of the injection interval (day 45, 48 and 52) was recommended, when they continued being completely symptom free.

**FIGURE 2:**
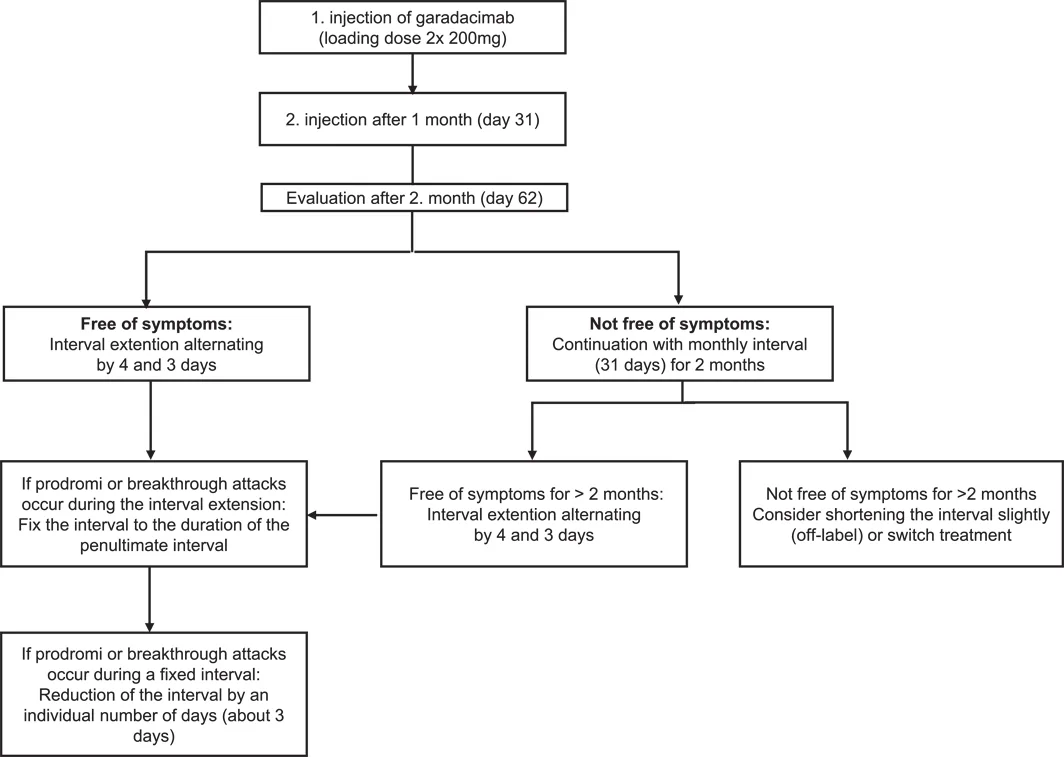
Berlin protocol for dosing interval individualization of garadacimab.

To ensure optimal adherence to the interval‐extension plan, all patients were provided with a schedule listing their individual projected injection dates. This schedule was created for a maximum injection interval of 63 days. Patients were asked to adhere to this plan until they noticed the first signs of HAE/AAE symptoms—either typical prodromal signs or even an attack. In the event of an attack, patients were instructed to treat it immediately with their ODT. As soon as any signs of HAE/AAE symptoms appeared, the interval‐extension protocol was stopped immediately, and the future treatment interval was set—following an in‐person or telephone consultation with our clinic—to at least the penultimate effective interval (but no shorter than once a month). This empirically determined individual interval represents the patient's future injection schedule. This new injection schedule remains in effect as long as the patient is completely symptom‐free during that interval.

### 2.3 Patient‐Reported Outcome Measures (PROMs)

Please see Supporting Information S1.

### 2.4 Statistics

Please see Supporting Information S1.

## 3 Results

### 3.1 The Prospect of Greater Comfort and Effectiveness Played a Major Role for Switching From Previous LTP to Garadacimab

For patients who were already using a different LTP before switching to garadacimab (*n* = 16), the major reason for previous users of berotralstat, pdC1INH and tranexamic acid was the prospect of improving the effectiveness of LTP. Two of three patients who administered pdC1INH also considered convenience with the treatment as reason for the switch. For the 11 patients previously treated with lanadelumab, the most commonly mentioned reason to switch was the prospect of more convenience. Specifically, nine patients cited convenience as a reason for switching, six cited efficacy, and six reported both reasons (Figure 3).

**FIGURE 3:**
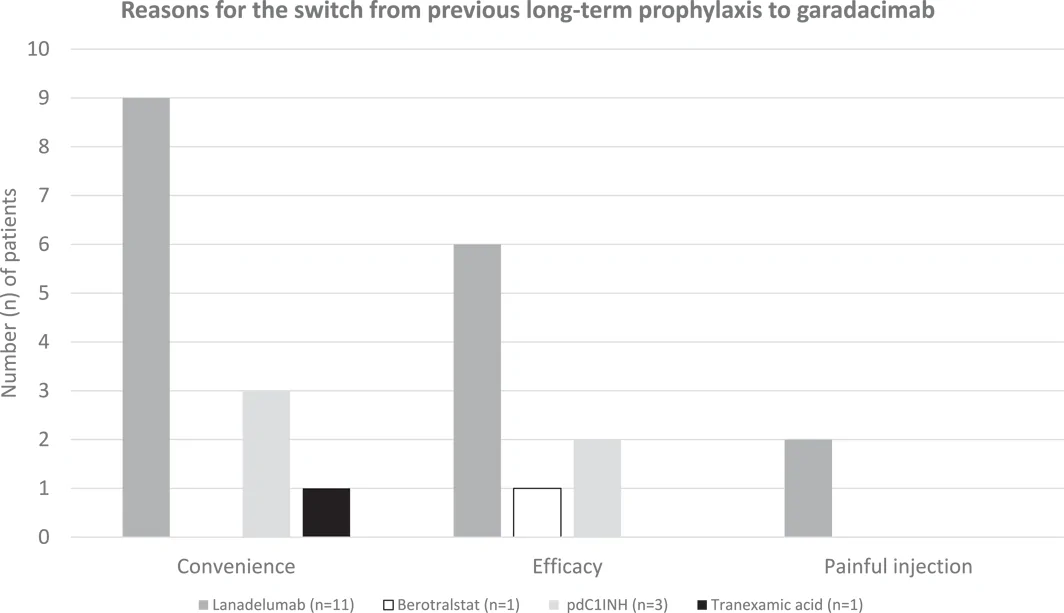
Reasons for the switch from previous long‐term prophylactic treatments, that is lanadelumab, berotralstat, plasma‐derived C1 inhibitor (pdC1INH) and tranexamic acid, to garadacimab.

Overall, four patients had previously received lanadelumab at 14‐day intervals, two at 21‐day intervals, one each at 28 and 32‐day intervals, and three at 35‐day intervals. All patients with previous lanadelumab injection intervals of < 31 days and two of three patients with injection intervals of 35 days mentioned convenience as the reason for the switch. Both patients, who had previously injected lanadelumab at intervals of 35 and 32 days, respectively, switched treatments due to injection site pain caused by lanadelumab. One patient with an injection interval of 35 days stopped lanadelumab to participate in a clinical trial, has been using ODT with icatibant in the meantime, however, finally he decided against participation and re‐initated LTP with garadacimab due to the outlook of improved convenience.

### 3.2 In Real‐Life, Most Patients Switched to Garadacimab at the end of the Individual Treatment Interval of the Previous Injectable LTP

Overall, patients with previous LTP with lanadelumab switched to garadacimab after a median period of 21 days (range 7–210) after the administration of their previous LTP, including two patients with very long intervals after the last lanadelumab injection: one due to non‐compliance (171 days, previously 32‐day interval) and one due to a washout attempt for participation in a clinical trial (210 days, previously 35‐day interval). The other patients with previous lanadelumab treatment switched at the end of the individual lanadelumab injection interval, which were 14 days (median, range 13–35 days), except one patient previously using a 35‐day dosing interval performed the switch seven days after his last injection.

Patients that previously used berotralstat and tranexamic acid switched on the next day; the one patient with HAE‐C1INH who used pdC1INH intravenously initiated garadacimab treatment 6 days after the last administration. One patient with AAE‐C1INH and previously receiving subcutaneous pdC1INH as LTP switched 20 days after her last injection (because her previous LTP supply was exhausted).

No breakthrough attacks were reported by patients during the transition from their previous LTP to garadacimab, with the exception of the two patients with HAE‐C1INH and the patient with HAE‐MYOF, in whom the attacks persisted and who discontinued treatment with garadacimab after the loading dose or the second dose, respectively, due to a perceived lack of efficacy.

### 3.3 Only a Few Patients Discontinued Garadacimab

At data cut, two female identical twins with HAE‐C1INH and the patient with HAE‐MYOF discontinued garadacimab after the loading dose and second dose, respectively.

The twins, who also both have comorbid chronic spontaneous urticaria treated with omalizumab, had previously managed their disease with lanadelumab at 14‐day injection intervals and at least weekly ODT on top, perceived insufficient efficacy of garadacimab and switched back to lanadelumab.

The patient with HAE‐MYOF did not report improvement after 2 months of treatment and discontinued garadacimab.

Due to the short treatment duration in these three patients and the lack of follow‐up data of PROMs, it was not possible to meaningfully evaluate these data, and therefore, these three patients were excluded from further analysis.

### 3.4 Garadacimab Injection Intervals Can Be Extended in Many Patients

Patients who continued garadacimab (*n* = 26) were observed for a median period of 26 weeks (time of data cut, range 9–48, *n* = 19, not including VANGUARD OLE‐RO patients).

Overall, the median injection interval of garadacimab (*n* = 26) at the time of data cut was 42 days (range 31–56 days). In all but three patients the approach of dosing interval prolongation was anticipated right from the start; in those in whom it was not anticipated, the decision against it was based primarily on convenience and/or efficacy concerns and these patients stayed at 31 day (monthly)—interval from the start. Of patients that agreed to extend the interval, 59% (*n* = 13) are still in the process of extending their individual dosing interval, whereas 10 patients have completed the interval extension due to the occurrence of prodromal signs and/or breakthrough attacks and are using fixed dosing intervals, including the five patients with monthly intervals. The current median dosing interval is 42 days (range 31–56) in patients still undergoing interval extension, and 47 days (range 31–49) for patients who, after attempting the dosing interval extension, are now continuing treatment with garadacimab at an individual, empirically determined dosing interval (Figure 4).

**FIGURE 4:**
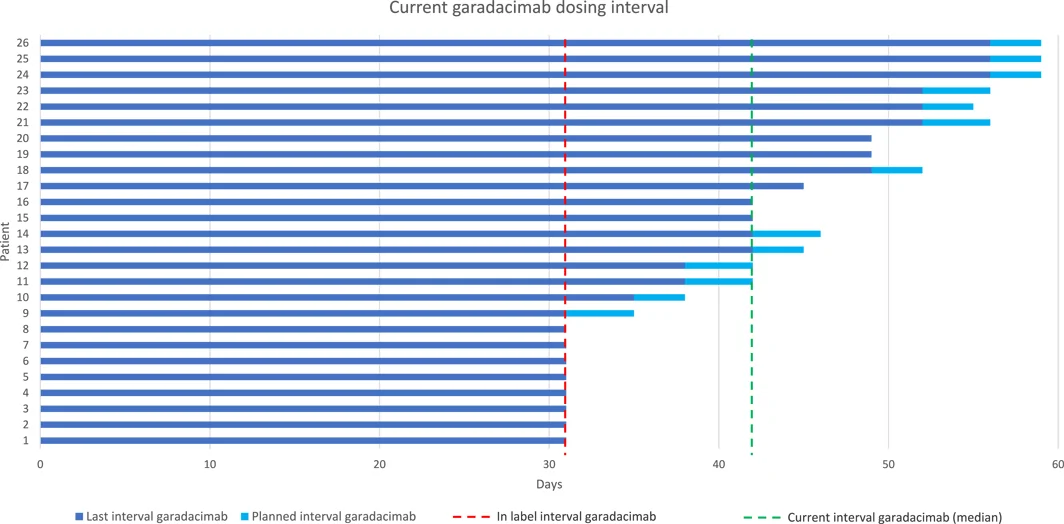
Current garadacimab dosing interval. In all but three patients the approach of dosing interval prolongation was initiated right from the start; in those in whom it was not initiated the monthly (= 31 days) dosing interval (in label, red line) was used from the start. Five patients initially aimed to prolong the interval from 31 to 35 days, but in the end used monthly dosing interval due to the occurrence of prodromal signs/breakthrough attacks. The green line indicates the current median dosing interval of the cohort.

### 3.5 Garadacimab Improves Disease Control in Almost all Patients Including Those who Have Previously Used Another LTP

Among all patients treated with garadacimab for whom data were available (*n* = 25), the mean AECT score was 14.5 points (SD 2.1, median 16) after a median duration of 25.7 weeks, and 96% and 40% achieved well‐controlled disease (≥ 10 points) and complete disease control (16 points), respectively.

Among the 20 patients with available AECT scores before and after switching to garadacimab, the score changed significantly after a median duration of 25.7 weeks from a mean of 9.2–14.4 points (paired *t*‐test, *p* < 0.001), with 14 patients (70%) achieving at least 3 points of improvement (minimal clinically important difference, MCID) and seven patients (35%) achieving complete disease control (16 points). The AECT score improved in all except three patients; however, in these three patients (one had AAE‐C1INH) the AECT showed well‐controlled disease before the switch (all previously used LTP with lanadelumab), and all remained in well‐controlled disease (Figure 5).

**FIGURE 5:**
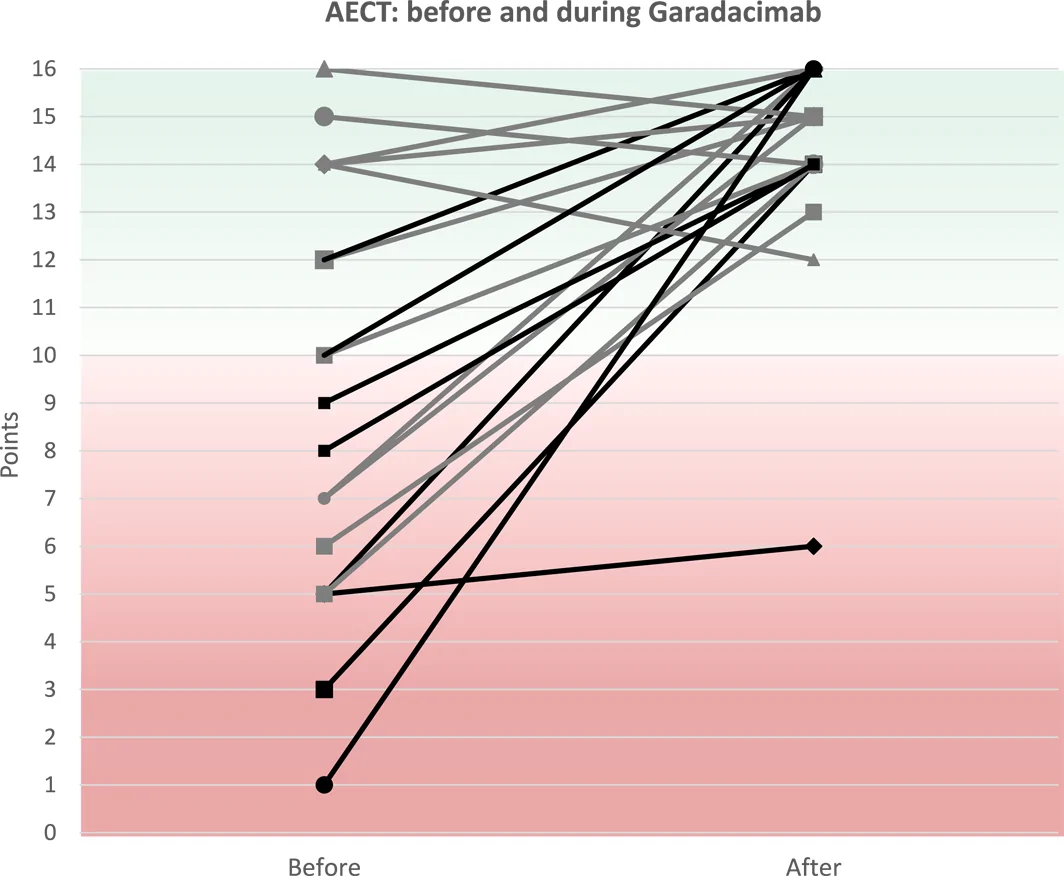
Angioedema Control Test (AECT) scores of 20 individual patients before and during treatment with garadacimab after a median treatment period of 25.7 weeks. Patients that switched from on demand treatment (ODT) are marked in black, patients that switched from other long‐term prophylaxis (LTP) are marked in gray. The cut‐off value of 10 points separates patients with insufficiently controlled disease (0–9 points, area highlighted in red) from those with well‐controlled disease (10–16 points, area highlighted in green).

Overall, patients who were using ODT before the switch showed lower mean AECT scores than patients with previous LTP at baseline, that is 6.7 points versus 11.2 points respectively. Furthermore, the improvement in AECT was numerically greater in patients who had previously received ODT, increasing from a mean of 6.7 (range 1–12) to 14.2 (range 6–16), compared with patients who had previously received other LTP (11.2 [range, 5–16] to 14.5 [range, 12–16]). The Welch two‐sample *t*‐test indicated a difference between groups (*p* = 0.034), whereas the Wilcoxon rank‐sum test yielded a borderline non‐significant result (*p* = 0.052). Given the small subgroup sizes and the sensitivity of the result to the statistical method applied, this exploratory finding should be interpreted with caution.

### 3.6 Garadacimab Improves Disease‐Related Quality of Life in Patients With HAE and AAE‐C1INH

The AE‐QoL was assessed in 25 patients under treatment with garadacimab after a median period of 25.7 weeks (range 9–48) and showed a mean score of 14 points. Twenty‐one patients (84%) had scores < 24, indicating no or negligible impact on quality of life, and eight patients had a score of < 10.

In 20 patients, AE‐QoL was available before and during treatment with garadacimab and the mean total score decreased significantly from 35.5 to 14.9 points (mean change score = 20.6 points, paired *t*‐test, *p* < 0.001). On average, the AE‐QoL score improved by more than 6 points (MCID) in both patient groups (ODT and LTP group) to garadacimab; patients switched from previous ODT had higher baseline AE‐QoL total scores and showed a greater improvement (from 43.1 to 15.7 points) than patients switched from previous LTP (from 29.4 to 14.3 points) (Figure 6).

**FIGURE 6:**
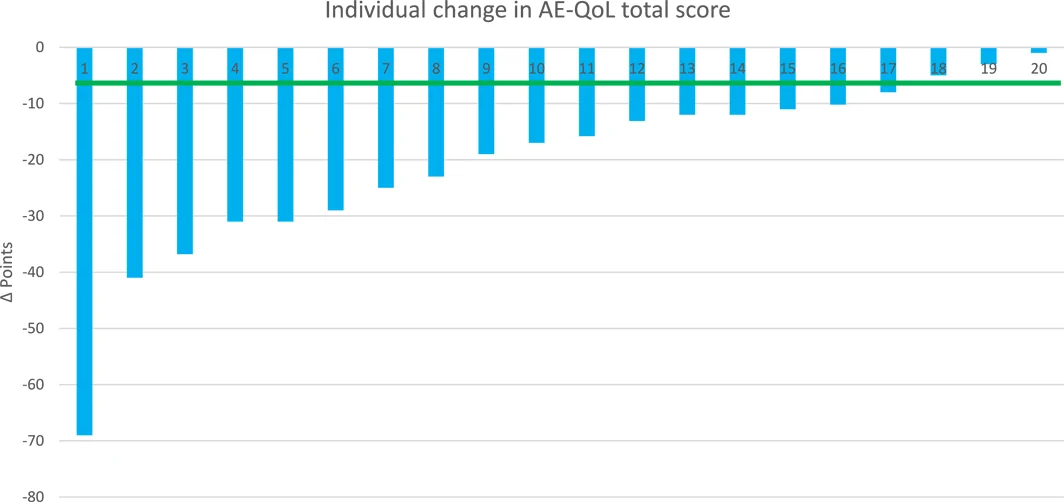
Individual change in the angioedema quality of life questionnaire (AE‐QoL) of 20 patients treated with garadacimab for a mean period of 25.7 weeks. Green line corresponds to the minimal clinical important difference (MCID) for improvement which is six points.

Overall, 85% (*n* = 17) achieved the MCID of at least 6 points for improvement in the AE‐QoL, and 82.4% (*n* = 14) of these patients simultaneously reached the MCID of 3 points in the AECT. The three patients that did not achieve MCID in AE‐QoL, among them one patient with AAE‐C1INH, already showed low AE‐QoL total values of ≤ 24 points at baseline, and switched from LTP with lanadelumab. All patients that switched from ODT and data available (*n* = 9 (6 patients from regular care as shown in Figure 1 and data from 3 patients prior to their participation in VANGUARD study)) achieved MCID in the AE‐QoL.

Within the individual domains of the AE‐QoL, we observed an average decrease of 29.2 (95% CI, 14.9–43.5; *p* < 0.001) in “functioning,” 13.4 (95% CI, 3.7–23.1; *p* = 0.010) in “fatigue/mood,” 22.3 (95% CI, 10.9–33.6; *p* < 0.001) in “fears/shame,” and 10.6 (95% CI, 0–21.3; *p* = 0.051) in the domain “nutrition”. The reductions in the individual domains were more pronounced in patients switched from ODT than in patients switched from other LTP (Figure 7).

**FIGURE 7:**
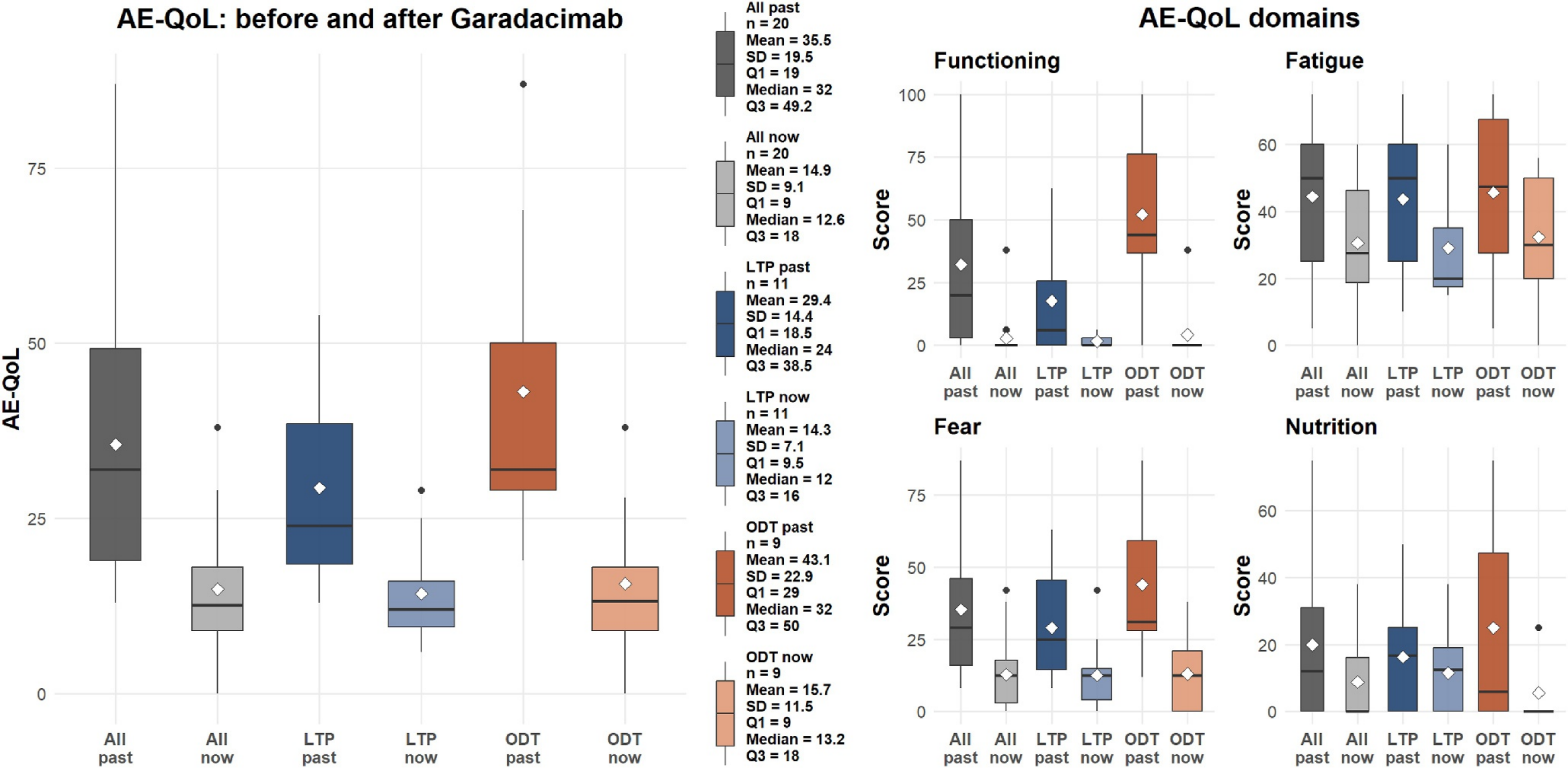
AE‐QoL total scores and domain scores (functioning, fatigue, fear/shame and nutrition) of patients before and during treatment with garadacimab (*n* = 20 patients) after a median treatment period of 25.7 weeks. AE‐Qol total scores before the switch to garadacimab are marked in dark gray, the AE‐QoL total scores after the switch in lighter gray. Patients that switched from ODT (ODT past and ODT now) are marked in orange, patients that switched from other long‐term prophylaxis (LTP), that is LTP past and LTP now, are marked in blue. Darker colors indicate scores before treatment, and lighter colors indicate scores during treatment. ODT: on‐demand treatment; LTP: long‐term prophylaxis.

### 3.7 Garadacimab is Well‐Tolerated

Safety data was documented from patients either via CARE or during the visit. All patients reported no side effects during treatment with garadacimab (i.e., answered “no” to the question in CARE: “In the last 3 months, did you have side effects from the currently used prophylactic medication?” or communicated this verbally during their last visit). In particular, patients found the injection handling with the pen and fine needle very pleasant.

## 4 Discussion

The therapeutic landscape for the management of HAE has changed significantly over recent years thanks to the many developments in this field. With the modern treatment options for LTP already available, the treatment goals for HAE, that is, the normalization of life and complete control of the disease, meaning freedom from signs and symptoms of HAE [9], became feasible for many patients, which has been demonstrated in clinical trials and real‐life observations in the past [1, 15, 16].

Findings from clinical trials and real‐life studies revealed, that the intended attack‐free status cannot be achieved for some patients using modern treatments for LTP, that is pdC1INH, berotralstat and lanadelumab [21]. It is therefore important and necessary to develop further options for LTP. Furthermore, in the development of new medications an increasing emphasis is being placed on convenience of administration, as this could reduce the burden of treatment and lead to an even better quality of life. For example, we previously observed, that, particularly with effective injectable LTP, the disease‐related quality of life of patients who were already well controlled improved further with longer injection intervals, that is 1 month and longer, which may be due to a reduction in the burden of treatment [22].

Garadacimab, which was launched in March 2025 in Germany, has not only demonstrated excellent efficacy and safety data but also, for the first time, the standard dosing required administration only once a month [14]. In the present study, we observed that the prospect of more convenience and efficacy with a new LTP were the most common reasons for patients of the cohort already using injectable LTP, that is lanadelumab, to switch to garadacimab via shared decision making. Even though it has been shown by us and others that many patients treated with lanadelumab in real life stay free from HAE attacks with injection intervals beyond 1 month (off‐label), there are patients who cannot achieve this but would prefer longer treatment intervals. In fact, all patients receiving lanadelumab at intervals of < 31 days cited convenience as a reason for the switch to garadacimab including 2 out of 3 patients previously using lanadelumab in a 35‐day interval. For only two patients, lanadelumab associated injection site pain, was the deciding factor for switching treatments.

To the best of our knowledge, this is the first real‐life observation of garadacimab in a larger cohort of HAE patients, including patients with AAE‐C1INH and HAE‐nC1INH with causative and unknown mutations. Our experience with garadacimab after 1 year confirms the efficacy and tolerability shown in controlled clinical trials in patients with HAE‐C1INH. Only three patients discontinued garadacimab due to perceived insufficient efficacy, among them two identical twins with HAE‐C1INH comorbid CSU and one patient with HAE‐MYOF, the latter case is a type of HAE described to be caused by vascular endothelial dysfunction, lanadelumab and/or berotralstat also were not effective and/or tolerated and the role of the kallikrein‐kinin‐system in this HAE type remains to be explored. Patients that continued garadacimab showed a clinical meaningful improvement in their disease‐related quality of life, disease control or both over a mean treatment period of 26 weeks while being very well tolerated, independent of their previous treatment (ODT or LTP). Overall, disease control improved in all patients who switched from previous ODT; in all except one patient by at least the MCID of 3 points. The patient with HAE‐FXII improved only by 1 point in her AECT (from 5 to 6 points), but showed clinical meaningful improvement in her QoL (AE‐QoL from 50 to 38 points). Particularly impressive was the response of the patient with HAE‐UNK, whose QoL and disease control improved significantly, that is from 87 to 18 points in the AE‐QoL and 1 to 16 points (complete control) in the AECT.

Among patients switched from previous LTP, AECT improved in all but three patients. These patients (one patient of them had AAE‐C1INH) had high baseline AECT scores (14–16 points) before the switch and were observed for a relatively short duration (median 11.9 weeks, range 11.4–34.1), but all remained in well‐controlled disease and improved in the AE‐QoL.

With regard to QoL, the improvement was even more pronounced: All patients improved and while all patients in the ODT group achieved an MCID of at least 6 points, this was the case for 81% of the patients who switched from previous LTP treatment.

Our study has the following limitations: In 6 patients, the comparative values for AECT or AE‐QoL before treatment with garadacimab were missing, particularly in those patients from VANGUARD‐OLE‐RO. Moreover, the study population is heterogeneous since it not only includes patients with HAE‐C1INH, but also patients with other entities, that is AAE‐C1INH and HAE‐nC1INH. Not least because of the small sample size, the relatively short observation period, and the fact that treatment response can vary across different disease entities, the generalizability of our results should be interpreted with caution.

The model used to extend the dosing interval is based, on the one hand, on previously published pharmacokinetic data for garadacimab [20], and, on the other hand, on a similar approach we previously described for lanadelumab in 2021 [15, 16]. We believe this approach effectively determines the longest individual dosing interval while also involving patients in the shared‐decision‐making process. Furthermore, as recently demonstrated with lanadelumab, the longer dosing intervals could lead to further improvements in QoL over time and increase patient satisfaction with the treatment [16, 22].

In this study, we showed that garadacimab injection intervals can be prolonged in many of the patients and that current median dosing interval is 11 days longer than the approved standard dosing interval (31 days = 1 month). Given the relatively short observation period to date (median 26 weeks) and the result that some patients had already reached dosing intervals of 56 days and were still in the extension phase, it is currently difficult to assess whether even longer dosing intervals are possible. Just a few weeks before this data collection was completed, donidalorsen—which can be administered once a month or every two months—became available in Germany for LTP of HAE attacks. It remains to be seen to what extent these treatments, as well as future treatment options with even longer injection intervals of 3 or 6 months, will impact on health‐related QoL [23].

In conclusion, this early real‐life observation confirmed the efficacy and safety of garadacimab as a new treatment option for LTP in HAE, while also demonstrating the potential to individualize the dosing interval according to the specific dosing needs of our HAE and AAE patients.

## Author Contributions

**Markus Magerl:** conceptualization, methodology, data curation, investigation, supervision, project administration, writing – original draft, writing – review and editing. **Kathrin Schön:** Data curation, investigation, project administration, writing – original draft, writing – review and editing. **Janine Ruhe:** investigation, writing – original draft. **Felix Aulenbacher:** methodology, data curation, formal analysis, visualization, writing – original draft. **Leonie Shirin Herzog:** investigation, writing – original draft. **Thomas Buttgereit:** conceptualization, methodology, data curation, investigation, project administration, writing – original draft, writing – review and editing.

## Funding

The authors have nothing to report.

## Ethics Statement

CARE was approved by Ethics Committee of Charité—Universitätsmedizin Berlin in August 2023 (EA2/145/23).

## Conflicts of Interest

Markus Magerl received travel grants and/or research funding and is or recently was a speaker and/or advisor for and/or has received research funding from Argo, Astria, Biocryst, Pharvaris, Takeda, CSL Behring, Otsuka, KalVista, and Intellia. Kathrin Schön has received travel grants and/or speaker/advisory fees from BioCryst, CSL Behring, KalVista, Pharvaris, Pharming and Takeda. Leonie Shirin Herzog has no conflicts of interest regarding this manuscript. Outside of this work LSH has received travel grants from Celltrion Healthcare and CSL Behring and was speaker for Celltrion Healthcare and Sanofi‐Aventis. Thomas Buttgereit received travel grants and/or research funding and is or recently was a speaker and/or advisor for Aquestive, AstraZeneca, Biocryst/Neopharmed Gentili, CSL‐Behring, Galderma, GlaxoSmithKline, Hexal, KalVista, Medac, Novartis, Otsuka, Pharming, Pharvaris, Roche, Sanofi, Swixx BioPharma and Takeda. Janine Ruhe and Felix Aulenbacher are declare no conflicts of interest.

## Supporting information


Supporting Information S1

## Data Availability

The data that support the findings of this study are available on request from the corresponding author. The data are not publicly available due to privacy or ethical restrictions.
