# Supplementary material for: Experiences From the First Year of Using Garadacimab to Treat Hereditary Angioedema and Acquired Angioedema
Source: Clin Transl Allergy. 2026 Oct 11;16(10):e70205. doi: 10.1002/clt2.70205 (PMC13654816; doi:10.1002/clt2.70205)
Supplement: Supplementary file 1 [file CLT2-16-e70205-s001.docx]

**Supplementary Material**

**Patient-Reported Outcome Measures (PROMs)**

All patients completed the Angioedema Control Test (AECT) and the Angioedema Quality of Life (AE-QoL) questionnaire before and after the switch to garadacimab.

The AECT is a validated instrument to assess patients’ disease control over recurrent angioedema symptoms retrospectively over the past four or 12 weeks. It contains four questions (Five answer options each, scored with 0-4 points) (16). The cut-off value of 10 points separates patients with insufficiently controlled disease (minimum 0 points) from those with well-controlled disease (maximum 16 points); the value for the minimal clinically important difference (MCID) is three points for improvement (17).

The AE-QoL is a validated questionnaire to assess angioedema-related QoL impairment in patients with recurrent angioedema. It contains 17 questions (Five answer options each, scored with 0-4 points) and encompasses the domains functioning, fatigue/mood, fears/shame and nutrition (18). The total score is calculated by using linear transformations of its raw scores to a 0 to 100-point scale. The MCID for improvement is 6 points; lower AE-QoL scores signify lower impairment in disease-related QoL(19).

**Statistical data analysis**

The current study is a retrospective observational real-life study, and thus no sample size was calculated. The most recent values of the individual AECT and AE-QoL scores under treatment with garadacimab were compared with the values before the switch. A paired 2-tailed t-test was performed to compare the AECT scores of the same participant before and after the switch. In addition, subgroup analyses according to previous treatment (ODT vs LTP) were performed for AECT, and change scores were compared between groups using a Welch 2-sample t-test. The Welch test was selected as the primary test because the analysis focused on differences in mean change scores and because it accounts for potentially unequal variances and group sizes. A Wilcoxon rank-sum test was additionally performed as a sensitivity analysis. Also, an exploration of individual injection intervals was conducted to monitor dose interval adjustments more accurately. The AE-QoL mean value before and during treatment as well as of each subdomain was determined, and it was assessed in which domain patients showed the most significant changes. For AE-QoL, paired 2-tailed t-tests were used to compare total scores before and during treatment, and mean changes with 95% confidence intervals and corresponding p values were calculated for the individual domains.
